# Epithelial-Mesenchymal Transition Stimulates Human Cancer Cells to Extend Microtubule-based Invasive Protrusions and Suppresses Cell Growth in Collagen Gel

**DOI:** 10.1371/journal.pone.0053209

**Published:** 2012-12-31

**Authors:** Jun Oyanagi, Takashi Ogawa, Hiroki Sato, Shouichi Higashi, Kaoru Miyazaki

**Affiliations:** 1 Graduate School of Integrated Science, Yokohama City University, Yokohama, Japan; 2 Division of Cell Biology, Kihara Institute for Biological Research, Yokohama City Universi, Yokohama, Japan; Ghent University, Belgium

## Abstract

Epithelial-mesenchymal transition (EMT) is a crucial event in tumor invasion and metastasis. However, most of past EMT studies have been conducted in the conventional two-dimensional (2D) monolayer culture. Therefore, it remains unclear what invasive phenotypes are acquired by EMT-induced cancer cells. To address this point, we attempted to characterize EMT cells in more physiological, three-dimensional (3D) collagen gel culture. EMT was induced by treating three human carcinoma cell lines (A549, Panc-1 and MKN-1) with TGF-ß. The TGF-ß treatment stimulated these cells to overexpress the invasion markers laminin γ2 and MT1-MMP in 2D culture, in addition to the induction of well-known morphological change and EMT marker expression. EMT induction enhanced cell motility and adhesiveness to fibronectin and collagen in 2D culture. Although EMT cells showed comparable cell growth to control cells in 2D culture, their growth rates were extremely suppressed in soft agar and collagen gel cultures. Most characteristically, EMT-induced cancer cells commonly and markedly extended invasive protrusions in collagen gel. These protrusions were mainly supported by microtubules rather than actin cytoskeleton. Snail-introduced, stable EMT cells showed similar protrusions in 3D conditions without TGF-ß. Moreover, these protrusions were suppressed by colchicine or inhibitors of heat shock protein 90 (HSP-90) and protein phosphatase 2A. However, MMP inhibitors did not suppress the protrusion formation. These data suggest that EMT enhances tumor cell infiltration into interstitial stroma by extending microtubule-based protrusions and suppressing cell growth. The elevated cell adhesion to fibronectin and collagen and high cell motility also seem important for the tumor invasion.

## Introduction

Like normal epithelial cells, ductal carcinoma cells *in situ* maintain cell polarity, which is supported by cell-cell contact and cell adhesion to basement membrane. During cancer progression, some carcinoma cells lose the cell polarity and invade through the basement membrane and then into connective tissue. This phenomenon is referred to as epithelial-mesenchymal transition (EMT) and thought to be a crucial event of cancer progression [1]–[3]. EMT is critical not only for many developmental steps such as gastrulation and neural crest formation but also for pathological events such as wound healing and tissue fibrosis [1], [3], [4]. EMT is generally characterized by the loss of epithelial marker E-cadherin, up-regulation of mesenchymal markers such as N-cadherin and vimentin, and acquisition of the fibroblast-like spindle cell shape in monolayer cultures [4].

EMT of cancer cells is induced typically by TGF-ß [5], but other growth factors and microenvironmental factors, such as HGF, EGF and FGF, are also able to induce or promote similar phenotypic changes depending on the cell types [1], [3], [6]. TGF-ß exerts multiple biological activities on the development and the growth, differentiation, extracellular matrix production and apoptosis of normal and cancer cells [7]. TGF-ß is a negative growth regulator of normal epithelial cells. Whereas TGF-ß suppresses tumor cell growth in early stages of carcinogenesis, it promotes tumor progression in later stages [7]. It has long been known that TGF-ß in a combination with other factors such as TGF-α and EGF, promotes anchorage-independent growth of normal fibroblasts [8]. The EMT-inducing activity of TGF-ß is mainly mediated by the Smad pathway, a major pathway of complex TGF-ß signals, which promotes expression of the EMT-related transcription factors including Snail, Slug, Twist, and ZEB 1/2 [6]. These transcription factors bind to E-box binding elements of promoter sequences and suppress expression of E-cadherin at a transcription level [9], [10].

EMT in cancer cells enhances the expression of invasion- or metastasis-related genes such as matrix metalloproteinases (MMPs). Our recent study showed that the tumor invasion marker laminin γ2, as well as MMP-9, is induced by the EMT induction of gastric cancer cells [11]. Other recent studies have suggested that EMT-induced cancer cells have resistance to anti-cancer drugs and radioactivity [12] and have cancer stem cell-like properties [13]. In spite of a number of past studies on the EMT of cancer cells, however, it is not clear how EMT contributes to tumor invasion and metastasis and what invasive phenotypes are acquired by EMT-induced cancer cells. This is at least in part owing to the fact that most EMT studies have been performed in the conventional two-dimensional (2D) culture system. It has become evident that cells grown in flat 2D culture considerably differ from those grown in three-dimensional (3D) culture in cell morphology, proliferation, differentiation, cell-cell interaction, cell-matrix interaction and gene expression [14], [15]. In order to understand the pathological consequence of EMT of cancer cells, it seems important to characterize EMT-induced cells in a more physiological 3D culture system.

In this study, we characterized EMT-induced cancer cells in both 2D monolayer culture and 3D collagen gel culture, using three cell lines. We found that EMT-induced cells showed prominent extension of microtubule-based invasive protrusions and growth suppression in the 3D collagen gel culture.

## Results

### EMT Induction of Three Human Cancer Cell Lines

**Figure 1:**
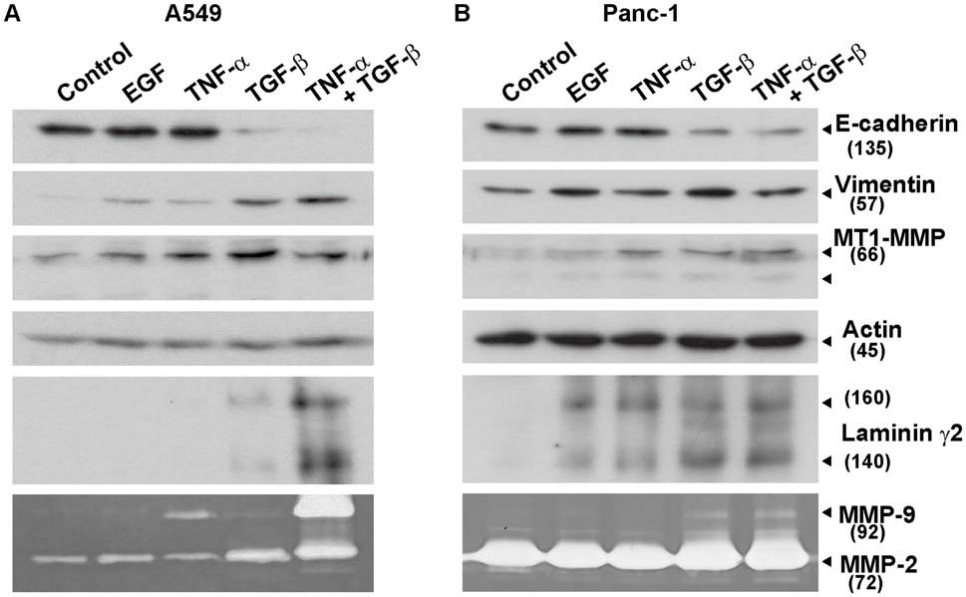
EMT induction in two cancer cell lines. A549 (A) and Panc-1 (B) cells were incubated in serum-free medium with 10 ng/ml EGF, 10 ng/ml TNF-α, 10 ng/ml TGF-ß, or TNF-α+TGF-ß. After 48-h incubation, conditioned media (CMs) and cell lysates were prepared and subjected to immunoblotting for EMT markers (E-cadherin and vimentin in the cell lysates), the invasion marker laminin γ2 (CMs), MT1-MMP (cell lysates), and actin as the internal loading control (cell lysates). The lowest panels show gelatin zymography of MMP-9 and MMP-2 in the CMs. Values in parentheses indicate approximate molecular size in kDa. Other experimental conditions are described in Materials and Methods.

To investigate phenotypic changes induced by EMT of cancer cells, we used models of three human cancer cell lines. We previously reported that TGF-ß and TNF-α synergistically induce EMT in serum-free culture of MKN-1 human gastric cancer cells [11]. It has been reported that lung adenocarcinoma cell line A549 [17] and pancreatic cancer cell line Panc-1 [16] undergo EMT by treatment with TGF-ß. In this study, we first tested three cytokines (TGF-ß, TNF-α, and EGF) for the EMT induction of A549 and Panc-1 cells, analyzing expression of some tumor invasion markers as well as EMT markers. In both cell lines, only TGF-ß was required to induce EMT as judged by the mesenchymal morphological change (Fig. S1), as well as reduction of E-cadherin expression and induction of vimentin (Fig. 1A and B). TGF-ß also simulated the expression of two tumor invasion markers, MT1-MMP and laminin γ2 chain, in both cell lines. However, a combination of TNF-α with TGF-ß further increased the levels of the laminin γ2 chain and MMP-9 in A549 cells (Fig. 1A). The laminin γ2 chain and MT1-MMP were also induced by TNF-α and/or EGF in Panc-1 cells (Fig. 1B). In addition, we found that although both TNF-α and TGF-ß were required for the EMT induction of MKN-1 cells in serum-free culture, only TGF-ß was necessary in the presence of serum (data not shown). These results indicate that TGF-ß is the most common and potent inducer of EMT for the three cancer cell lines, but TNF-α is also required for efficient expression of some invasion markers, depending on the cell types.

### Effect of EMT Induction on Cell Adhesion and Migration in Monolayer Culture

**Figure 2:**
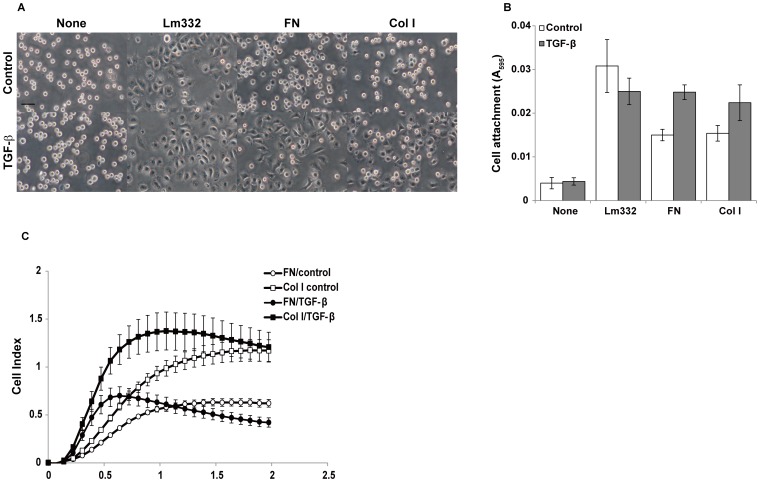
Effect of EMT induction on adhesion activity of A549 cells. (A and B) A ninety-six-well plate was coated with 2 µg/ml laminin-332 (Lm332), 5 µg/ml fibronectin (FN), and 2 µg/ml collagen I (Col I) at 4°C overnight, and then blocked with 1.2% BSA for 1 h at 37°C. A549 cells were incubated with 10 ng/ml TGF-ß in serum-free medium for 24 h. The TGF-ß-treated cells (TGF-ß) and untreated cells (Control) were suspended and inoculated onto the plate. After incubation for 30 min, phase contrast images were taken (A) and cell adhesion activity was measured (B). A scale bar indicates 50 µm. The relative numbers of attached cells were determined as described in Materials and Methods. Each bar indicates the mean ± SD of adherent cells in triplicate wells. (C) Electric time-lapse cell adhesion assay with E-plates. The plate was precoated with fibronectin (○, •) and collagen I (□, ▪) as shown above, and the adhesion of TGF-ß-treated cells (TGF-ß: •, ▪) and untreated cells (Control: ○, □) to the plate was monitored every 5 min. Cell index indicates arbitrary unit reflecting attachment and spreading of the cells on microelectrode array in the bottom of the wells. Other experimental conditions are described in Materials and Methods.

As shown above, TGF-ß efficiently induced EMT in the three different carcinoma cell lines. Next, we examined what phenotypes were acquired by the EMT induction of cancer cells for their invasive growth. First, the cell adhesion activity of A549 cells was investigated after EMT induction, using three cell adhesion substrates, laminin-332, fibronectin and type I collagen. The cells were pre-treated with TGF-ß for 24 h and then seeded on these substrates. As shown in Figs. 2A and 2B, untreated control cells most efficiently adhered to laminin-332. TGF-ß treatment only slightly decreased the cell adhesion to laminin-332. In contrast, it significantly increased the cell adhesion efficiency to the stromal substrates fibronectin and type I collagen. The electric time-lapse cell adhesion assay clearly showed the EMT-induced changes in the cell adhesion activity of A549 cells to fibronectin and type I collagen (Fig. 2C). These changes were also observed in Panc-1 and MKN-1 cells (data not shown).

**Figure 3:**
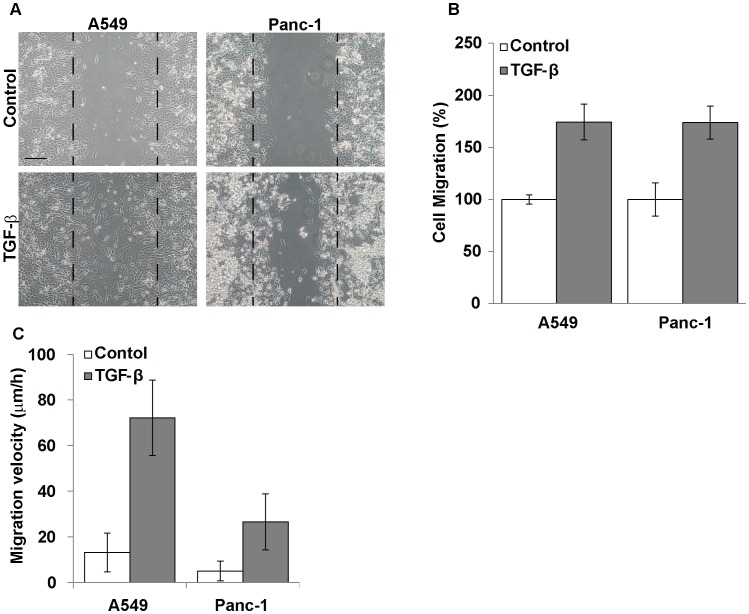
Migration activity of EMT-induced cancer cells. (A and B) To measure cell migration activity, A549 (left panels) and Panc-1 (right panels) cells were subjected to the *in vitro* wound healing assay in the presence or absence (Control) of TGF-ß, as described in the text. Phase-contrast micrographs of the cultures were taken after 16-h incubation. Black broken lines indicate initial wound edges. Scale bar, 50 µm. B) Cell migration area was estimated by analyzing the pixel with Image J. Each bar indicates the mean ± SD for the areas of migrated cells in 3 different fields (right panels). (C) Cells that had been pretreated without or with TGF-ß for 24 h were incubated in 1% FBS-containing medium without or with TGF-ß onto a 24-well plate for 6 h at 37°C. The cell migration was monitored with a time-lapse video system for 12 h. Cell migration distance was measured for 15 randomly selected cells. Each bar indicates the mean ± SD for cell migration velocity of 15 cells. Other experimental conditions are described in Materials and Methods.

Secondly, cell migration activity was investigated using two different assays. In *in vitro* wound healing assay, TGF-ß significantly promoted cell migration of A549 and Panc-1 cells in the absence of TNF-α under serum-supplemented conditions (Fig. 3A and B). TNF-α did not have additional effect on the cell migration activity (data not shown). When cell migration was assayed by the time-lapse video microscopy, the cell motility enhanced by the TGF-ß treatment was more clearly shown with the two cell lines (Fig. 3C). We also confirmed that the TGF-ß treatment increased the motility of MKN-1 cells in the wound healing assay (data not shown).

### Effect of EMT Induction on Anchorage-dependent and -independent Cell Growth

**Figure 4:**
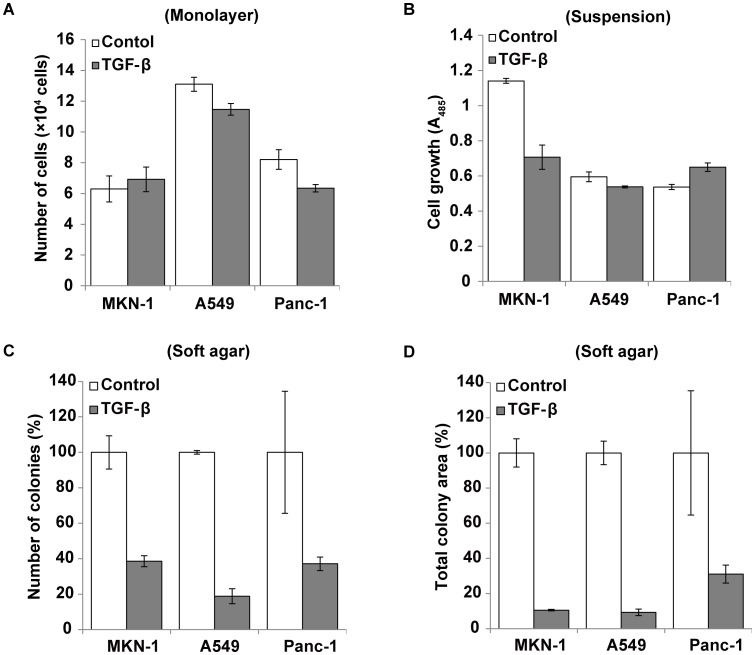
Growth of three EMT-induced cancer cell lines in three different conditions. (A) MKN-1 (1.0×10^4^ cells), A549 (0.5×10^4^ cells) and Panc-1 (1.0×10^4^ cells) were incubated in each well containing 1% FBS-containing medium without (Control) or with TGF-ß on 24-well culture plates for 7 days in monolayer culture. After the incubation, the number of cells was measured with a cell counter. Each bar indicates the mean ± SD of the cell numbers in triplicate wells. (B) Cells were cultured at a density of 1×10^4^ cells per well in 24-well suspension culture plates containing 1% FBS-containing medium without (Control) or with TGF-ß for 7 days. The relative number of cells was measured using Dojindo cell counting kit 8. Each bar indicates the mean ± SD of the absorbance values at 485 nm in triplicate wells. (C and D) Cells were inoculated at a density of 7,000 cells per 35-mm dish in 10% FBS-containing soft agar medium without (Control) or with TGF-ß and incubated for 10 (MKN-1) or 14 (A549 and Panc-1) days. After the incubation, cells were stained with *p*-iodonitrotetrazolium violet, and the number of total colonies (C) and total colony area (D) were determined by Image J and shown as the relative number to the control (100%). Each bar indicates the mean ± SD in triplicate wells. Other experimental conditions are described in Materials and Methods.

Thirdly, cell growth activity of EMT-induced cells was investigated under three different conditions. In 2D monolayer culture, TGF-ß treatment did not affect the growth of MKN-1 cells and negligibly or only slightly suppressed that of A549 and Panc-1 cells (Fig. 4A). When colony formation was examined at sparse 2D cultures (500 cells/35-mm dish) of A549 and Panc-1 cells, there was no significant difference between the TGF-ß–treated and non-treated cultures (data not shown). In suspension culture on non-adhesive plates, the three cell lines slowly grew forming cell aggregates or spheroids on the plates. Under such conditions, the TGF-ß treatment suppressed the growth of MKN-1 cells but had no effect on that of A549 and Panc-1 cells (Fig. 4B). In contrast, the colony formation of the three cell lines in soft agar culture was strongly suppressed by the TGF-ß treatment. Both the number of total colonies and the total colony area were extremely reduced in the TGF-ß-treated cell lines than the non-treated ones (Fig. 4C, 4D, and S2). However, the TGF-ß-treated Panc-1 cells formed relatively large colonies compared to the non-treated cells (Fig. S2). The lack of E-cadherin-mediated cell-cell adhesion in TGF-ß-treated cells might suppress the potentials of cellular survival and proliferation in the anchorage-independent condition.

### Behavior of EMT-induced Cancer Cells in 3D Collagen Gel Culture

**Figure 5:**
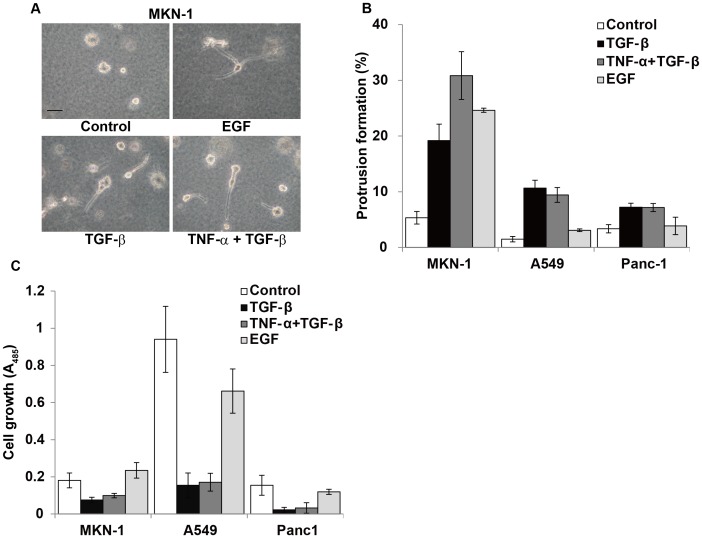
Morphological change and growth of EMT-induced MKN-1 cells in 3D collagen gel. (A) MKN-1 cells were incubated in 3D collagen with or without indicated cytokines on 3-well chamber slides for 7 days. Culture medium was changed every 3rd day. Scale bar, 50 µm. (B) After 5-day incubation, the numbers of total cell clumps and ones with protrusions were counted in a center field under a microscope, and the percentage of protrusion-positive cell clumps was calculated. Each bar indicates the mean ± SD of the relative numbers of protrusion-positive cell clumps in triplicate wells. (C) After 7-day incubation, the cells in collagen gel were stained with Dojindo cell counting kit 8 for 4 h, and the absorbance at 485 nm of each culture medium was measured. Each bar indicates the mean ± SD of the absorbance values in triplicate wells. Other experimental conditions are described in Materials and Methods.

Gene expression *in vivo* is different from that in the monolayer culture system (15). As a culture system that mimics *in vivo* conditions, we used 3D collagen gel culture to further characterize the EMT-induced cancer cells. Cancer cells were cultured within collagen gel layer containing TGF-ß and/or other factors for 7 days. In the 3D collagen gel, MKN-1 cells showed prominent membrane extensions or protrusions when treated with TGF-ß (Fig. 5A and B). A similar morphological change was observed in A549 and Panc-1 cell lines (Fig. S3). The addition of TNF-α to the TGF-ß-containing culture further promoted this morphological change only in the case of MKN-1 cells (Fig. 5B). EGF alone, which induced EMT in MKN-1 cells, also induced the protrusion formation in this cell line, but this effect was not observed in A549 and Panc-1 cells. These results suggest that the morphological change is specific for EMT induction rather than TGF-ß stimulation.

We next examined whether EMT induction suppressed cell growth within collagen gel. As found in soft agar culture, TGF-ß strongly suppressed the growth of all cell lines examined in collagen gel (Fig. 5C). EGF never or only weakly suppressed the growth of the three cell lines in 3D collagen gel. TNF-α did not have any significant additional effect in these cultures. These data indicate that the EMT induction promotes prominent extension of protrusions but suppresses cell growth in the 3D collagen gel cultures of the three cancer cell lines.

### Microtubule-based Structure of EMT-induced Protrusions in 3D Collagen Gel

**Figure 6:**
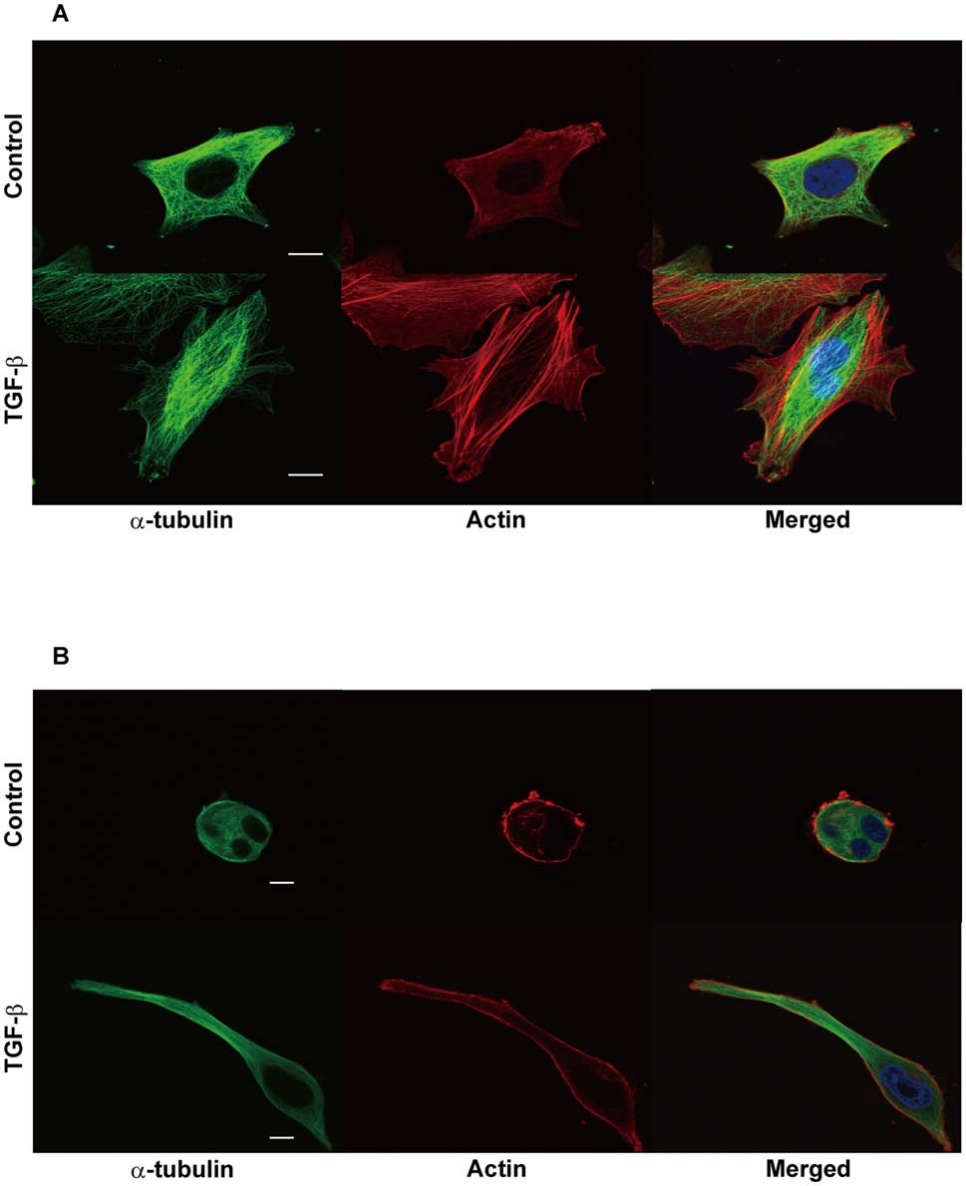
EMT-induced change of cytoskeletal structure of cancer cells cultured in monolayer (2D) and 3D collagen gel cultures. MKN-1 cells were incubated without (Control) or with 10 ng/ml TGF-ß in serum-containing medium in 2D culture (A) or 3D collagen gel culture (B) for 3 days. These cells were fluorescence-stained for α-tubulin (green) by using a specific antibody, F-actin by rhodamine phalloidin (red), and DAPI (blue). Fluorescence images were obtained with a confocal fluorescence microscope. Other experimental conditions are described in Materials and Methods.

To characterize the EMT-induced protrusions, we analyzed cytoskeletal changes after EMT induction by TGF-ß. Filamentous actin (F-actin) and microtubule cytoskeletons of EMT-induced cells were stained by fluorescence with rhodamine phalloidine and a tubulin-specific antibody, respectively. MKN-1 cells were stimulated with TGF-ß in 2D and 3D cultures and then stained for the cytoskeletons (Fig.6A and B). In the 2D culture of EMT-induced cells, robust stress fibers of F-actin, as well as microtubules, supported the unique cell shape (Fig. 6A). In the 3D collagen gel, F-actin filaments were strongly detected at the surface of spheroid structure in unstimulated cells (Fig. 6B). In TGF-ß-treated cells, peripheral actin filaments were found around the cell body and protrusions, but stress fibers were rarely found in the protrusions. In contrast, many bundles of microtubules were prominently detected in the center of the protrusions in the EMT-induced cells. In control cells, microtubules were evenly distributed in the cytoplasm. These patterns of cytoskeletons were commonly found in MKN-1 and Panc-1 cells (data not shown).

**Figure 7:**
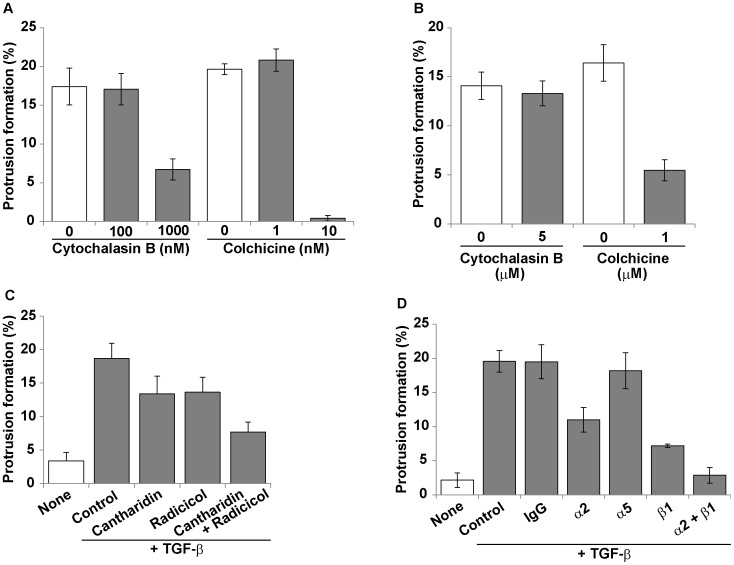
Effects of various inhibitors on protrusion formation of MKN-1 cells in 3D collagen gel. MKN-1 cells were incubated with 10 ng/ml TGF-ß in serum-containing medium in 3D collagen gel culture, as described in Figures 5 and 6. To the collagen culture were added various inhibitors, and the protrusion formation was quantified 24 h later (A, C and D) or 3 h later (B). (A) The indicated concentrations of cytochalasin B and colchicine were added into the culture medium at the same time as the TGF-ß addition. (B) Cytochalasin B (5 µM) and colchicine (1 µM) were added into the culture medium after incubation with TGF-ß for 24 h. (C) MKN-1 cells were treated without (Control) or with cantharidin (1 µM) and/or radicicol (1 µM) as described in (A). (D) MKN-1 cells were pretreated with 10 µg/ml of each neutral antibody at 37°C for 30 min, and the pretreated cells were embedded into collagen gel containing 10 µg/ml of the indicated anti-integrin antibody and 10 ng/ml TGF-ß. All these inhibitors were not cytotoxic at least under the above experimental conditions, as analyzed by the trypan blue staining. However, cytotoxic effects became evident when the cells were incubated with 5 µM cytochalasin B or 1 µM colchicine for 24 h.

To examine whether microtubule is a principle cytoskeleton supporting the EMT-induced protrusions, we examined effects of some cytoskeletal inhibitors on the EMT-induced cell extension in collagen gel. Cytochalasin B and colchicine are known to inhibit both polymerization and stabilization of F-actin and microtubules, respectively. When the cells were treated with these inhibitors at the same time as the TGF-ß treatment, the cell extension was partially inhibited by 1 µM cytochalasin B, but completely by 10 nM colchicine (Fig. 7A). When these inhibitors were added 24 h after TGF-ß stimulation, the protrusions were significantly retracted by colchicine after 3-h treatment, but not by cytochalasin B (Fig. 7B). Similarly, two other microtubule inhibitors vinblastin and taxol (paclitaxel) dose-dependently blocked the cell extension when added together with TGF-ß to the 3D culture (Fig. S4). We also analyzed effects of some signal inhibitors on the EMT-induced cell extension in collagen gel. The protein phosphatase 2A (PP2A) inhibitor cantharidin and the heat shock protein-90 (HSP-90) inhibitor radicicol, both which inhibit the stabilization of microtubules, weakly inhibited the cell extension (Fig. 7C). When both cantharidin and radicicol were added in combination, the cell extension was additively suppressed. These data suggest that the protrusions of EMT-induced cancer cells in 3D collagen gel are mainly supported by microtubules, and both PP2A and HSP-90 activities are necessary for the protrusion formation. Although actin cytoskeleton is required for the extension of protrusions, it seems unnecessary for maintaining the protrusion structures.

The protrusions formed in EMT-induced cancer cells within collagen gel greatly differed in appearance from the cell morphology in the monolayer culture. This implies that the interaction of the cells with collagen in the 3D environment is involved in the protrusion formation. This possibility was examined by using anti-integrin, neutral antibodies. As expected, anti-integrin-α2 and anti-integrin-ß1 antibodies effectively blocked the extension of protrusions (Fig. 7D). A combination of the two antibodies more strongly inhibited the protrusion formation than each antibody. These results demonstrate that the formation of microtubule-based protrusions requires both EMT-inducing cytokine and collagen/integrin signals.

#### Differences from other invasive protrusions

It is well known that malignant cancer cells invade into 3D extracellular matrix by extending some kinds of protrusion or projection. Invadopodium is an actin-based, typical protrusion produced by invasive cancer cells [18]. MT1-MMP is thought to be a marker of invadopodia and its activity is required for invadopodium formation. To test whether the protrusions observed in the EMT-induced cancer cells inside the collagen gel are identical to invadopodia, we examined effect of a broad spectrum MMP inhibitor (TAPI) on the protrusion extension. When TAPI was simultaneously added with TGF-ß into the collagen culture, little or very weak effect on the protrusion extension was obtained in the three cell lines tested (Fig. S5, A–C). Essentially the same results were obtained when TIMP-2, a natural MMP inhibitor, was used instead of TAPI (data not shown).

Because Src kinase activity is also associated with the invadopodium formation, we next examined effects of three kinds of Src kinase inhibitors, PP1 analog, SU6656 and lavendustin C, on the protrusion formation in 3D collagen gel. Any kinds of the inhibitors did not suppress the protrusion formation of MKN-1 cells (Fig. S5, D). These results suggest that the EMT-induced protrusion is a different cell structure from invadopodium.

### Microtubule-based Protrusions in Snail-induced EMT Cells within Collagen Gel

**Figure 8:**
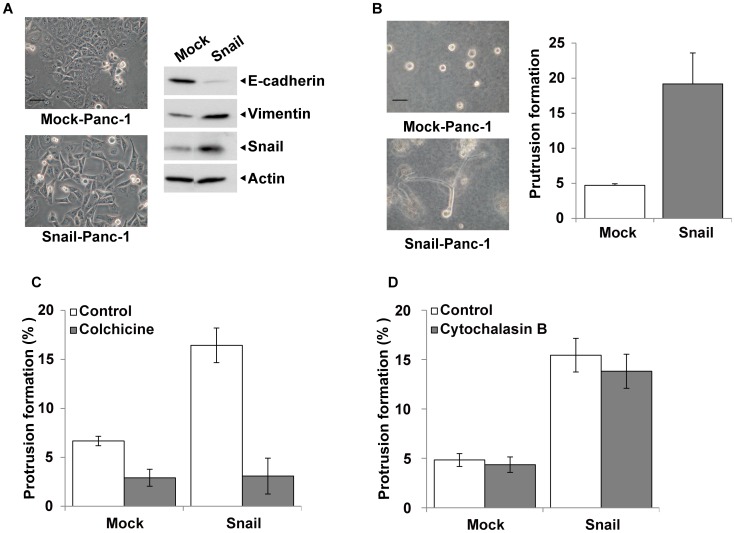
Effect of constitutive EMT-induction in Panc-1 cells on protrusion formation in 3D collagen gel. Panc-1 cells were transfected with an empty vector (Mock-Panc-1) or a snail expression vector (Snail-Panc-1), and their stable transfectants were established. These cells were cultured without TGF-ß in 2D monolayer culture or 3D collagen gel culture. (A) Morphology of Mock-Panc-1 (upper panel) and Snail-Panc-1 (lower panel) cells incubated for 2 days in 2D monolayer culture, and expression of E-cadherin, vimentin and snail in these cells (right panels). See Figure 1 for experimental conditions. Scale bar, 50 µm. (B) Morphology of Mock-Panc-1 (upper panel) and Snail-Panc-1 (lower panel) cells incubated in 3D collagen gel culture for 3 days, and their protrusion formation (right figure). See Figure 5 for experimental conditions. Scale bar, 50 µm. (C and D) Effects of 1 µM cholchicine (C) and 5 µM cytochalasin B (D) on protrusion formation in 3D collagen gel culture. These inhibitors were added into the culture medium after 24-h incubation with TGF-ß. Other experimental conditions are the same as described in Figures 5 and 7B.

As shown above, TGF-ß-stimulated cancer cells commonly extend microtubule-based invasive protrusions in the 3D collagen gel culture. To more generalize this phenomenon, we established stably EMT-induced cells by introducing a snail cDNA expression vector into Panc-1 cells (Snail-Panc-l). Like TGF-ß-stimulated cells, Snail-Panc-l cells showed scattered cell morphology as compared with the empty vector-transfected, control cells (Mock-Panc-1) (Fig. 8A). In accordance with the morphological change, E-cadherin expression was suppressed and vimentin expression was enhanced in Snail-Panc-l cells as compared with the control cells (Fig. 8A). When these transfected cells were seeded into 3D collagen gel, Snail-Panc-l but not Mock-Panc-1 cells extended microtubule-based, robust protrusions in the absence of TGF-ß (Fig. 8B). The extension of protrusions in Snail-Panc-l cells was strongly inhibited by colchicine but scarcely by cytochalasin B (Fig. 8C and D). These data also support that the microtubule-based protrusion formation reflects EMT in 3D collagen gel.

**Figure 9:**
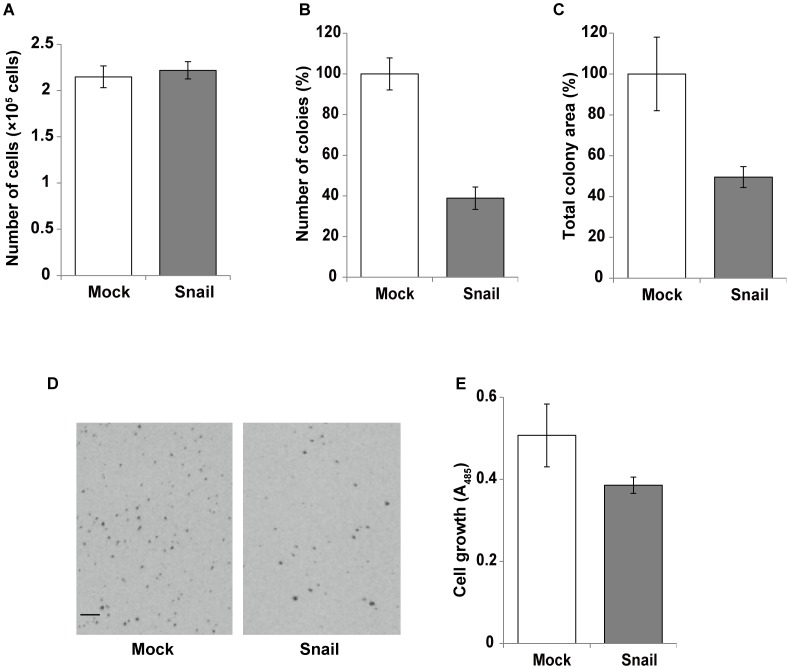
Growth of Snail-expressed Panc-1 cells in three different conditions. (A) Monolayer culture. Mock-Panc-1 (open column) and Snail-Panc-1 (closed column) were seeded at a density of 1.0×10^4^ cells per well of 24-well plates in 10% FBS-containing medium and incubated for 7 days. After the incubation, the number of cells was measured with a cell counter. (B–D) Soft agar culture. Mock-Panc-1 and Snail-Panc-1 cells were cultured in soft agar medium for 14 days. After the incubation, the number of total colonies (B) and total colony area (C) were analyzed by Image J. The soft agar cultures were photographed and each typical image is shown in (D). Scale bar, 500 µm. Other experimental conditions were the same as described in Figure 4. (E) Collagen culture. Mock-Panc-1 and Snail-Panc-1 cells were cultured in 3D collagen gel for 7 days as described in Figure 5. After the incubation, the relative number of cells was measured using Dojindo cell counting kit 8.

We also compared the growth potentials in anchorage-dependent and independent conditions between Mock-Panc-1 and Snail-Panc-l cells. There was no significant difference in growth rate between the two kinds of cells in monolayer culture (Fig. 9A). In soft agar culture, both total colony number and total colony area were clearly lower in Snail-Panc-l cells than Mock-Panc-1 cells (Fig. 9B and C), but large colonies were more abundant in Snail-Panc-l cells than the control cells (Fig. 9D). The cell proliferation in collagen gel was slightly but significantly lower in Snail-Panc-l cells than the control cells (*p*<0.05) (Fig. 9E).

## Discussion

In this study, we used EMT models of three human carcinoma cell lines (A549, Panc-1 and MKN-1) to characterize the EMT-induced cells in both 2D and 3D culture systems. In agreement with other studies [16], [17], A549 and Panc-1 cells exhibited typical EMT phenotypes after treatment with TGF-ß alone in 2D monolayer cultures. MKN-1 cells required TGF-ß plus TNF-α in serum-free medium for EMT induction as reported previously [11], but only TGF-ß was required in serum-containing medium. Expression of the two invasion markers laminin γ2 and MT1-MMP was associated with the EMT induction of these cell lines. However, expression of MMP-9 and the laminin γ2 was much greater with TGF-ß plus TNF-α than TGF-ß alone [11]. These results suggest that the acquisition of invasive phenotypes of cancer cells requires TNF-α and/or other factors besides TGF-ß, even if TGF-ß alone is enough for the morphological EMT induction.

The present study revealed that EMT-induced A549 cells more efficiently adhered to the stromal cell adhesion substrates fibronectin and type I collagen than uninduced control cells, though the cell adhesion to the basement membrane substrate laminin-332 was not changed by the EMT induction. This is consistent with the fact that expression of stromal extracellular matrix proteins such as fibronectin and type I collagen is enhanced by EMT induction [16]. We also confirmed the increased fibronectin production in the EMT-induced cells in 2D culture (data not shown). It is highly expected that the EMT-induced change of cell adhesion activity depends on that of integrin expression. Furthermore, we showed that the cell migration potentials of the three cell lines in 2D culture were significantly increased by the EMT induction, in agreement with the results for Panc-1 cells reported by Horiguchi et al. [17]. These phenotypic changes, which are consistent with the general concept of EMT, seem to be required for cancer cell invasion through interstitial stromal tissues.

The present study revealed great differences in the phenotypes of EMT-induced cancer cells between 2D and 3D cultures. Firstly, the cell growth in response to TGF-ß was completely different between the two culture systems. The TGF-ß treatment did not have significant effect on the cell growth in monolayer and suspension cultures of the three cell lines, except for the suppression of the MKN-1 cell growth in suspension. In contrast, the growth of the three cell lines in soft agar and collagen gel cultures was strongly inhibited by the TGF-ß treatment. TGF-ß is a potent growth suppressor of normal epithelial cells and cancer cells at an earlier stage, whereas it promotes their progression at a later stage [7]. The canonical TGF-ß-Smad pathway suppresses cell growth through up-regulating p15 and p21 CDK inhibitors, but non-smad signal is also involved in the growth suppression through PP2A activity [19]. However, Ras signal activation overcomes growth suppressive effect of TGF-ß [20]–[22]. Moreover, the Ha-Ras-TGF-ß combination promotes invasive and metastatic behavior of cancer cells [23], [24]. A549 and Panc-1 cells have already acquired a mutation that constitutively activates Ras [25], [26]. MKN-1 cells are also resistant to TGF-ß because its receptor expression is very low [27]. These facts may explain the lack of growth suppression by TGF-ß in the 2D cultures of these cell lines. However, constitutive activation of Ras might be insufficient for escaping from the growth suppression by TGF-ß when these cells are placed as single cells into soft agar or collagen gel. Because E-cadherin-mediated cell-cell adhesion generates a cell survival signal, the loss of E-cadherin and cell-cell contact by the EMT induction is likely to reduce the anchorage-independent growth ability of cells. In addition, it seems reasonable to consider that invading cancer cells transiently lose their cell proliferation activity in 3D matrix. However, our results do not necessarily imply that cancer cells with mesenchymal phenotypes always have suppressed proliferation activity in 3D conditions. TGF-ß was originally found as a cytokine that enhances the anchorage-independent growth of normal fibroblasts in the presence of TGF-α or EGF (8). Therefore, it seems possible that EMT-induced cancer cells restore the anchorage-independent growth activity by the expression or presence of some growth factors and cell adhesion proteins *in vitro* and *in vivo*. It is noted that the stably EMT-induced cell line Snail-Panc-1 showed relatively high growth activity in collagen gel as compared with TGF-ß-treated Panc-1 cells. This implies that even if the original cells are identical, the two types of EMT-induced cells have somewhat different phenotypes with respect to the growth potential in 3D conditions.

Secondly, we found that the cell morphology of EMT-induced cells in 3D collagen gel completely differed from that in 2D monolayer culture. The EMT induction stimulated the three cancer cell lines to extend prominent invasive protrusions in 3D collagen gel culture. The protrusion formation was promoted by TNF-α plus TGF-ß more strongly than TGF-ß alone in MKN-1 cells. EGF alone, which is able to induce EMT in MKN-1 cells [11], also stimulated the cell extension in this cell line but not the others. This suggests that the cell extension is associated with EMT induction rather than the TGF-ß activity. The invasive protrusions were mainly supported by microtubules, and its formation was blocked by colchicine. Although integrin-dependent actin cytoskeleton was required for the generation of the invasive protrusions, it seemed unnecessary for the maintenance of the protrusions. When mammalian cells receive extracellular stimuli, they dynamically change their structures, extending pseudopodia or protrusions. These include filopodia and lamellipodia, both which are essential structures for cell migration and observed mostly in 2D cultures [28]. Invadopodium and podosome, which are thought to be very similar or identical to each other, are well known as invasive structures of cancer cells [28], [18]. All these structures are actin-based structures. The formation or function of podosomes and invadopodia require MT1-MMP, Src kinase, PI3 kinase and other signal or cytoskeletal regulators [29]–[31]. The cell protrusion found in this study is a microtubule-based structure, and its shape and size are clearly different from the above mentioned cell structures. Furthermore, inhibitors for Src kinase and PI3K did not inhibit the invasive protrusions in 3D collagen gel. Therefore, it may be concluded that the EMT-induced invasive protrusion is a different structure from the four actin-based cell structures. Recent studies have demonstrated some similarities between EMT-induced cells and cancer stem cells (12, 13). Like tissue stem cells, cancer stem cells exist in a dormant or quiescent state in special microenvironments called niches [32]. This is consistent with our finding that EMT-induced cells have suppressed growth activity in 3D collagen gel. However, it is unlikely that cancer stem cells actively migrate through 3D matrix, extending protrusions. There seem to be important differences between EMT-induced cancer cells and cancer stem cells.

Recently, Whipple et al. [33], [34] reported a novel microtubule-based protrusion ‘microtentacle’, which is induced in suspension by EMT. The microtubule-associated protein tau is essential for the microtentacle formation [35]. In our study, PP2A and HSP-90, both which regulate microtubule stability by acting on tau [36], [37], were also essential for the protrusion formation in 3D collagen gel. The invasive protrusions in this study required integrin-mediated interaction with collagen and greatly differed from microtentacles in appearance. Many recent studies have investigated the mechanism of cell invasion within 3D collagen gel or 3D matrix. For example, invasive human cancer cells such as HT1080 fibrosarcoma and MDA-MB-231 breast carcinoma cells invade through collagen gel by projecting a long membrane extension [38], [39]. The long protrusions of MDA-MB-231 cells are supported by microtubules [39]. Although the mesenchymal cell invasion requires MMP and Src activities, the presence of MMP inhibitors allows them to migrate through the collagen matrix by exhibiting an amoeboid cell structure [38]. However, the MMP requirement may depend on the cross-linkage of collagen fibers used in experiments [40]. It is also well known that when MDCK cells are treated with HGF in collagen gel, they start to invade the collagen gel, generating a membrane extension [41]. A recent study has shown that the invasive structure of MDCK cells is supported by microtubules [42]. Similarly, Ha-Ras-transformed mammary epithelial cells form tubular structures in collagen gel by TGF-ß treatment [23]. These invasive structures seem to be very close or essentially identical to the invasive protrusions of the EMT-induced cancer cells found in the present study. It has been reported that signal mediators for actin cytoskeleton, e.g. PI3K-Akt signal [43] and Rho-GTPases [44], [45], also regulate microtubule stability, and TGF-ß activates Rho and Rac signals [46], [47]. In the present study, however, neither PI3K-Akt signal inhibitors nor a ROCK inhibitor suppressed the protrusion formation. These discrepancies suggest substantial differences in cytoskeletal regulation between 2D and 3D conditions. Further studies are required for clarifying how TGF-ß induces the microtubule-based invasive protrusions in 3D collagen gel.

In conclusion, our study demonstrates that EMT-induced cancer cells acquire a mesenchymal phenotype and invade into collagen matrix by projecting robust microtubule-based protrusions. EMT cells lose cell proliferation potential in collagen matrix. This suggests that mesenchymal-epithelial transition (MET) under a different microenvironment is required for the cancer cells to grow again. Our findings provide a clinical clue to suppress tumor invasion *in vivo*.

## Materials and Methods

### Antibodies and Reagents

Mouse monoclonal antibody (mAb) against the human laminin γ2 chain (D4B5) was established and characterized previously [48]. Other antibodies used and their source are as follows: mouse mAb against human E-cadherin from Becton Dickinson (Franklin Lakes, NJ), mouse mAb against human vimentin from Sigma Aldrich (St. Louis, MO); mouse mAb against human α-tubulin and function-blocking antibodies against integrins α2 (P1E6), α5 (P1D6) and ß1 (6S6) from Chemicon (Temecula, CA); rabbit polyclonal antibody against human actin from Biomedical Technologies (Stoughton, MA), and FITC-conjugated anti-mouse IgG antibody from Vector laboratories (Burlingame, CA). Human transforming growth factor-ß1 (TGF-ß1), human epidermal growth factor (EGF), human tumor necrosis factor-α (TNF-α), colchicine, cytochalasin B, and SB431542 were purchased from Wako pure chemical (Osaka, Japan), and radicicol and cantharidin from Calbiochem (La Jolla, CA).

### Cell Culture and Preparation of Conditioned Media and Cell Lysates

Human adenocarcinoma cell lines of the stomach (MKN-1) and lung (A549) were provided from Japanese Collection of Research and Bioresources (JCRB, Tokyo, Japan) in 1993 and stored in liquid nitrogen after a few passages [48]. MKN-1 and A549 cells secrete laminin-332 and laminin-511, respectively. Their laminin production was confirmed in this study. Pancreatic adenocarcinoma cell line Panc-1 was provided from JCRB in 2004. Its identity was confirmed in this study from similarity in the response to TGF-ß to that in a previous study [16]. These cell lines were cultured in DMEM/F12 medium (Invitrogen, Carlsbed, CA) supplemented with 10% fetal bovine serum (FBS) (Nichirei Biosciences, Tokyo) at 37°C in a humidified atmosphere of 5% CO_2_ and 95% air. This medium was used as the standard serum-containing medium unless otherwise noted. Conditioned media were prepared as reported previously [11]. Cells were grown to confluence in serum-containing medium. After the incubation, the cultures were further incubated in serum-free medium with or without cytokines for 2 days. The resultant conditioned media were collected, dialyzed against distilled water and lyophilized. The dried protein was dissolved in a 1/50 volume of distilled water. Cells were lysed in RIPA buffer (20 mM Tris-HCl, pH7.5, 150 mM NaCl, 5 mM EDTA, 2.5 mM tetra-sodium pyrophosphate, 1 mM sodium orthovanadate, 10 mM NaF, a protease inhibitor mixture (Wako), 1% Nonidet P-40, 0.5% sodium deoxycholate, and 0.1% SDS) and centrifuged at 10,000×g for 10 min. The resultant supernatants were used as cell lysates.

### Electrophoretic Analyses

SDS-PAGE was performed on 6 or 10% polyacrylamide gels under non-reducing or reducing conditions. Immunoblotting of EMT marker proteins and gelatin zymography of MMPs were carried out as reported previously [11].

### Cell Adhesion Assays

Cells were incubated at a density of 2.0×10^4^ cells per well in 0.1 ml serum-free medium for 30 min at 37°C on 96-well microtiter plates, which had been precoated with each cell adhesion substrate and blocked with 1.2% (w/v) bovine serum albumin (BSA). After non-adherent cells were removed by gentle agitation, adherent cells were fixed with 2.5% glutaraldehyde and stained with 0.5% (w/v) crystalviolet for 30 min at room temperature. Each well was measured for absorbance at 595 nm with Plate Chameleon V (Hydex; Turka, Finland). Alternatively, time-lapse cell adhesion was electrically analyzed using the xCELLigence System Real-Time Cell Analyzer (Roche; Basel, Switzerland), according to the manufacture’s instruction manual. E-plate was coated with each substrate protein and blocked with BSA. Cell attachment was measured every 5 min.

### Cell Migration Assays

Confluent cultures were incubated with 10 ng/ml TGF-ß in serum-containing medium for 24 h and then with 10 µg/ml mitomycin C for 2 h to prevent cell growth. The cell monolayer was scratched with a 200 µl pipette tip, washed with PBS, and incubated in fresh DMEM/F12 plus 1% FBS with or without TGF-ß for 16 h. Phase-contrast micrographs were taken immediately and 16 h after wounding. The scratched area was analyzed by Image J. In random cell migration assay, cells stimulated by TGF-ß for 24 h were inoculated in 1% FBS-supplemented medium with or without TGF-ß. After 6-h incubation, cell migration was monitored at 37°C with a time-lapse video system for 12 h. The distance of cell migration was measured by using a video monitor (Olympus VM-30; Tokyo).

### Cell Growth Assays

For the assay in monolayer culture, cells were incubated on 24-well culture plates in DMEM/F12 plus 1% FBS supplemented with or without 10 ng/ml TGF-ß. After incubation for 7 days, grown cells were harvested and counted with a cell counter (Sysmex CDA-500; Hyogo, Japan). For suspension culture, cells were incubated in the same medium as above on 24-well Celltight X suspension culture plates (Sumilon; Tokyo). After incubation for 7 days, the relative number of grown cells was determined with the cell counting kit 8 (Dojindo; Kumamoto, Japan). For soft agar culture, cells were incubated in 0.33% (w/v) agar medium containing 10% FBS with or without TGF-ß, which was overlying 1% (w/v) agar layer in 35-mm dishes. The cultures were incubated at 37°C for 10 or 14 days. Colonies were visualized by incubating with 0.5 mg/ml of *p*-iodonitrotetrazolium violet for 24 h, and the total number and area of colonies in a center field were analyzed by Image J.

### Collagen Gel Culture

Collagen gel culture was performed using bovine type I collagen (Nitta Gelatin, Osaka, Japan) on 3-well assay slides (AR Brown, Tokyo, Japan). A base layer of 2.1 mg/ml collagen in serum-containing medium (30 µl) was prepared in each well. Thirty µl of cell suspension containing 15,000 cells was mixed with 120 µl of the unsolidified collagen gel solution, and its 30-µl aliquot was placed on the base layer. After 30-min incubation at 37°C, the upper gel was overlaid with 100 µl of serum-containing medium with or without growth factors, and incubated for 1 week. The medium was replaced every 3rd day. Cell growth was assessed using the cell counting kit 8.

### Immunofluorescent Staining of Cells in Collagen Gel

Cells cultured in collagen gel for 3 days were fixed in 10% formalin in PBS for 1 h and washed 3 times with PBS. The cells were permeabilized with 0.1% (v/v) Triton X-100 in PBS (PBST) for 1 h, blocked with 5% BSA in PBS for 2 h, and incubated overnight at 4°C with the primary antibody against α-tubulin diluted with 5% BSA in Triton X-100/PBS. The cells were then stained with FITC-labeled secondary antibody against mouse IgG, rhodamine phalloidin, and DAPI for 3 h at room temperature. The fluorescence image was observed using a confocal fluorescence microscope (Olympus FV1000-D BX61; Tokyo).

### Construction of Snail Expression Vector and Transfection

Snail cDNA clone (pF1KB7546) was purchased from Kazusa DNA research institute (Chiba, Japan). pF1KB7546 was digested with SpeI and SmaI, and cloned into a Xba-SmaI site of the mammalian expression vector pBOS-CITE-NEO to make pBOS-CITE-NEO-snail [11]. Panc-1 cells were transfected with an empty vector (Mock-Panc-1) or the snail expression vector (Snail-Panc-1) using Xtreme gene 9 transfection reagents (Roche, Basel, Switzerland), and their stable transfectants were selected with 500 µg/ml of G418 (Calbiochem; La Jolla, CA).

## Supporting Information

Figure S1 **EMT induction in two cancer cell lines, A549 and Panc-1.** A549 and Panc-1 cells were incubated with 10 ng/ml EGF, 10 ng/ml TNF-α, 10 ng/ml TGF-ß, or TNF-α+TGF-ß in serum-free medium. After incubation for 48 h, phase contrast micrographs were taken. x 300.
(TIF)

Figure S2 **Colony formation of three cell lines in soft agar cultures with or without TGF-ß.** MKN-1, A549, and Panc-1 cells were incubated in soft agar medium with or without TGF-ß. After incubation for 14 days, the cultures were photographed. Each typical image is shown. Other experimental conditions were the same as described in Fig. 4C.
(TIF)

Figure S3 **Morphological change of EMT-induced cancer cells in 3D collagen gel.** A549 (A) and Panc-1 (B) cells were incubated in 3D collagen with or without indicated cytokines on 3-well chamber slides for 7 days.
(TIF)

Figure S4 **Effects of paclitaxel and vinblastin on protrusion formation of MKN-1 cells in 3D collagen gel.** MKN-1 cells were incubated without (open columns) or with the indicated concentrations of paclitaxel (A) or vinblastin (B) 10 µM TAPI-1 in the presence of TGF-ß for 5 days, and protrusion formation was quantified. Other experimental conditions are described in Figures 5 and 7.
(TIF)

Figure S5 **Effects of synthetic MMP inhibitor and signal inhibitors on protrusion formation of MKN-1 cells in 3D collagen gel.** MKN-1(A), A549 (B), and Panc-1 (C) cells were incubated without (open columns) or with 10 µM TAPI-1 in the absence (Control) or presence (TGF-ß) of TGF-ß for 5 days, and protrusion formation was quantified. (D) MKN-1 cells were incubated without (None) or with the following inhibitors in the absence (open column) or presence (closed columns) of TGF-ß for 24 h: PP1-analog (1 µM), SU6656 (1 µM), or lavendustin C (1 µM). Other experimental conditions are described in Figures 5 and 7.
(TIF)
