# Supplementary figures and images for: Epithelial-Mesenchymal Transition Stimulates Human Cancer Cells to Extend Microtubule-based Invasive Protrusions and Suppresses Cell Growth in Collagen Gel
Source: PLoS One. 2012 Dec 31;7(12):e53209. doi: 10.1371/journal.pone.0053209 (PMC3534040; doi:10.1371/journal.pone.0053209)

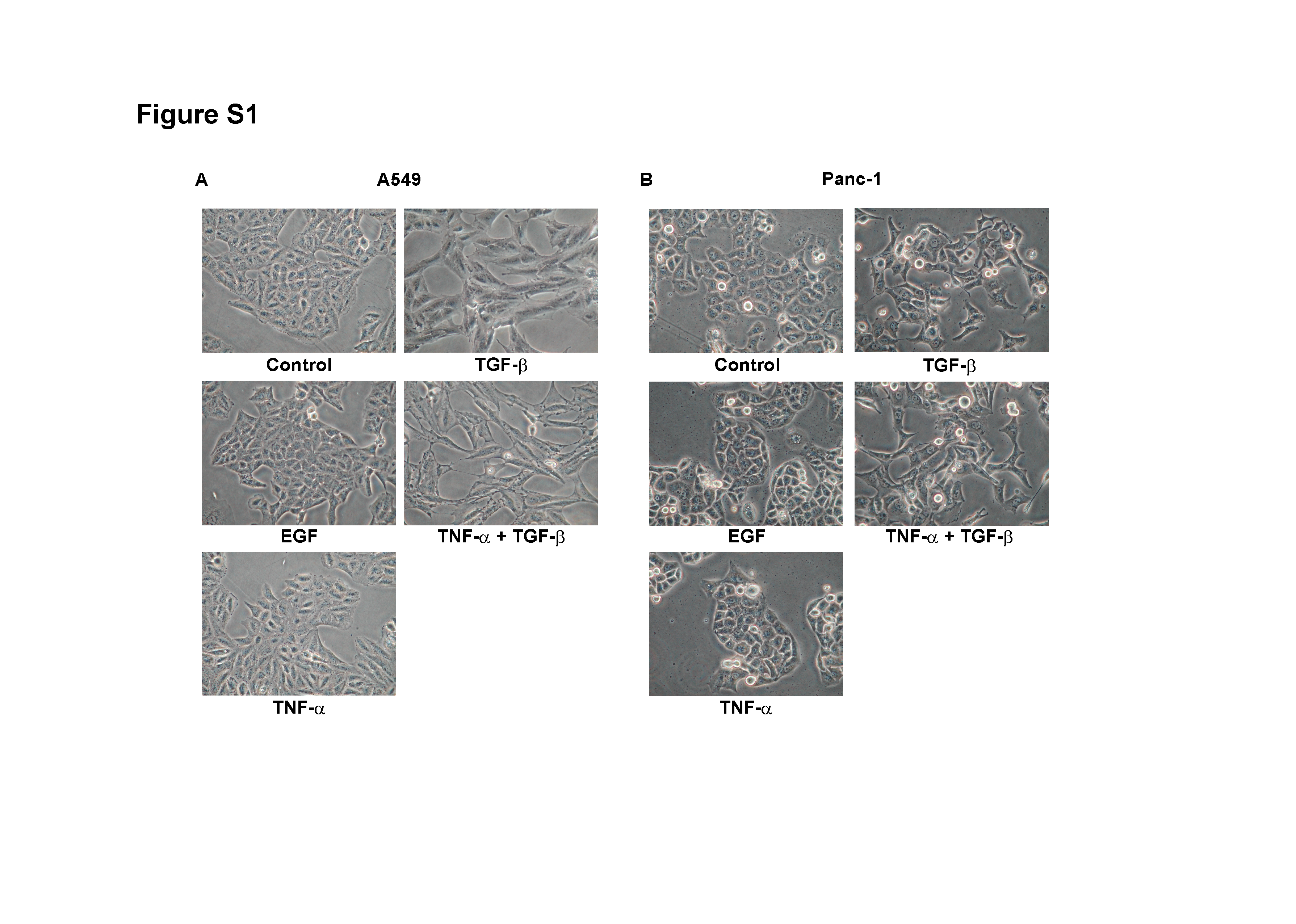

Supplement: Figure S1 — EMT induction in two cancer cell lines, A549 and Panc-1. A549 and Panc-1 cells were incubated with 10 ng/ml EGF, 10 ng/ml TNF-α, 10 ng/ml TGF-ß, or TNF-α+TGF-ß in serum-free medium. After incubation for 48 h, phase contrast micrographs were taken. x 300. (TIF) [file pone.0053209.s001.tif]

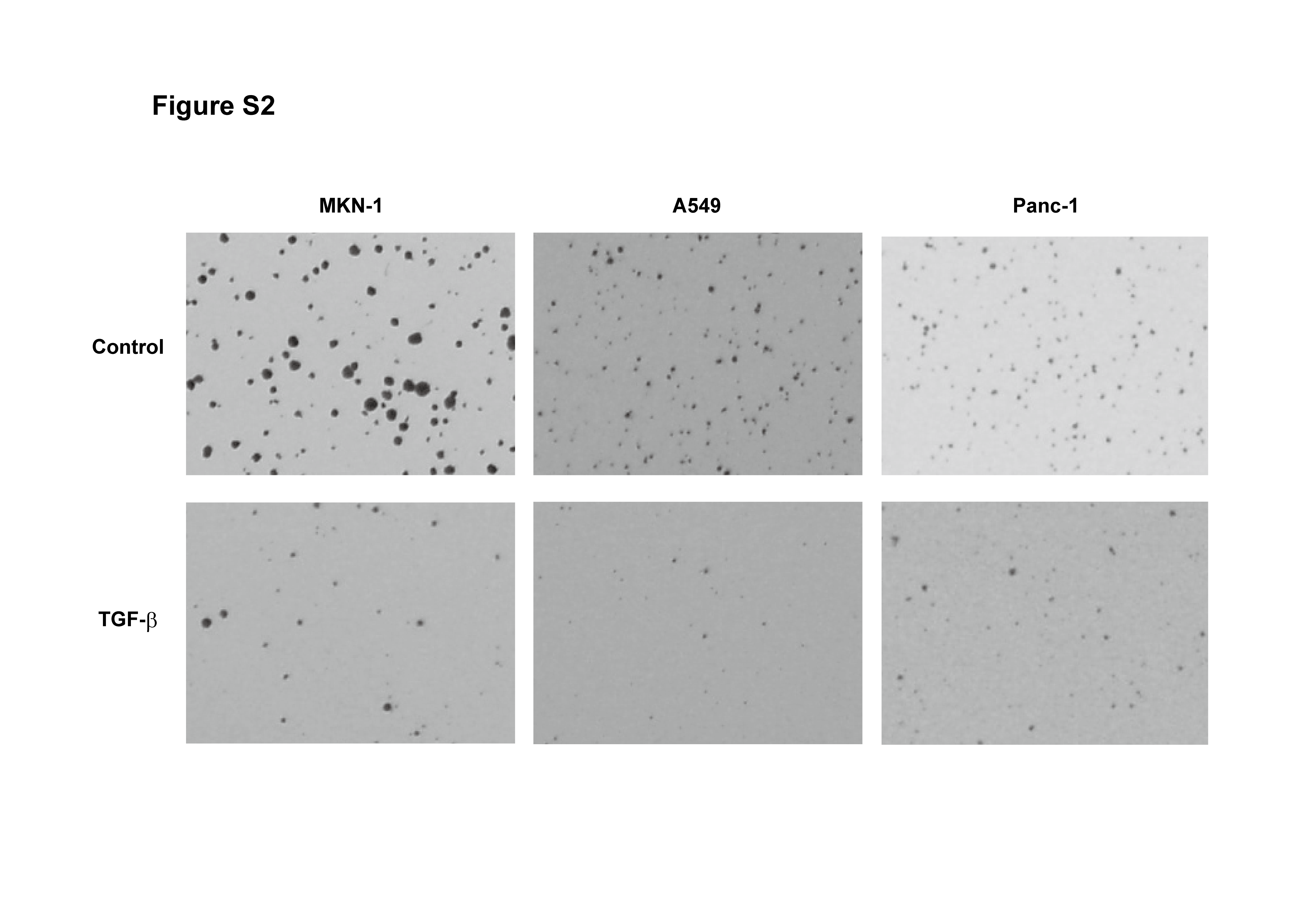

Supplement: Figure S2 — Colony formation of three cell lines in soft agar cultures with or without TGF-ß. MKN-1, A549, and Panc-1 cells were incubated in soft agar medium with or without TGF-ß. After incubation for 14 days, the cultures were photographed. Each typical image is shown. Other experimental conditions were the same as described in Fig. 4C. (TIF) [file pone.0053209.s002.tif]

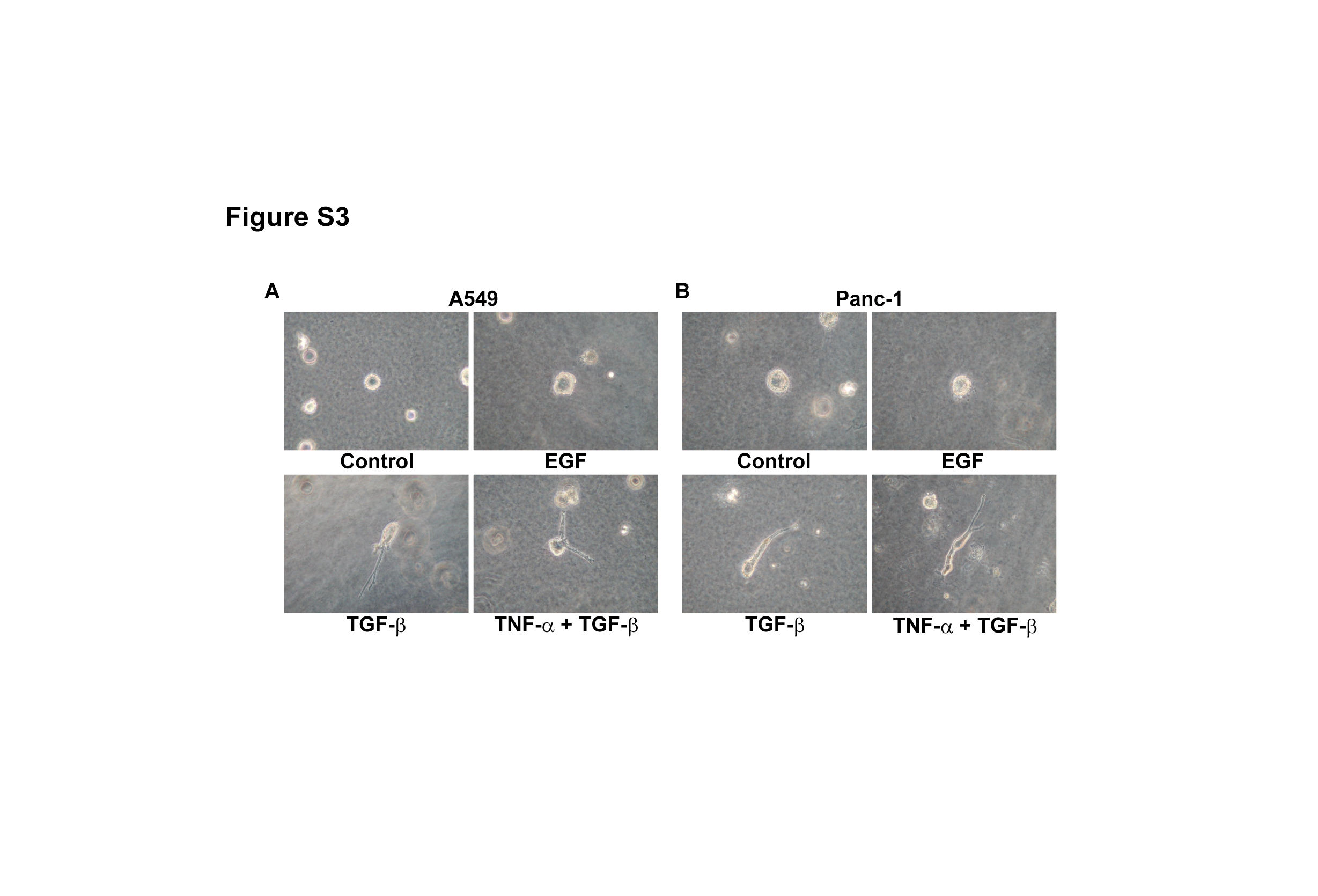

Supplement: Figure S3 — Morphological change of EMT-induced cancer cells in 3D collagen gel. A549 (A) and Panc-1 (B) cells were incubated in 3D collagen with or without indicated cytokines on 3-well chamber slides for 7 days. (TIF) [file pone.0053209.s003.tif]

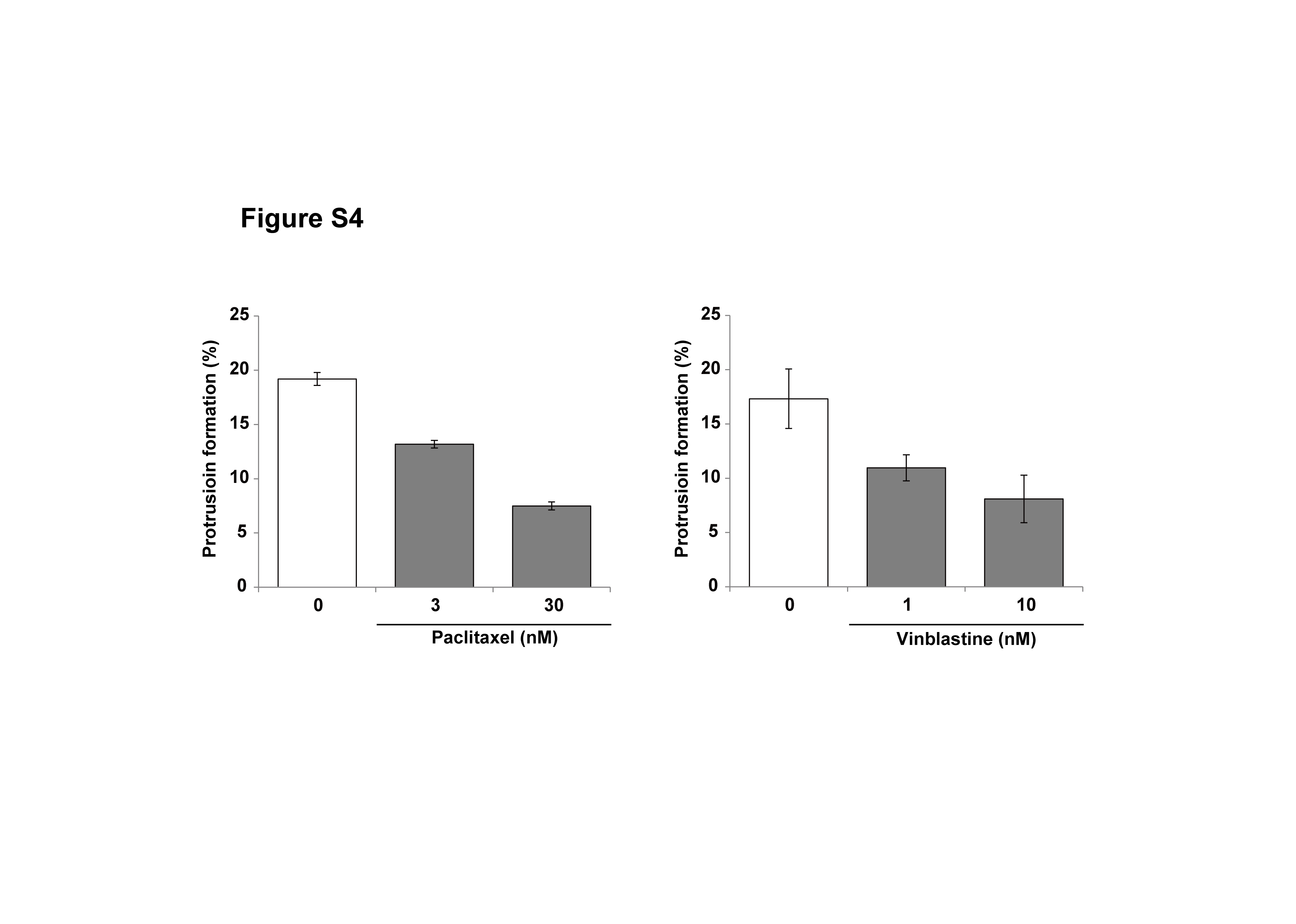

Supplement: Figure S4 — Effects of paclitaxel and vinblastin on protrusion formation of MKN-1 cells in 3D collagen gel. MKN-1 cells were incubated without (open columns) or with the indicated concentrations of paclitaxel (A) or vinblastin (B) 10 µM TAPI-1 in the presence of TGF-ß for 5 days, and protrusion formation was quantified. Other experimental conditions are described in Figures 5 and 7. (TIF) [file pone.0053209.s004.tif]

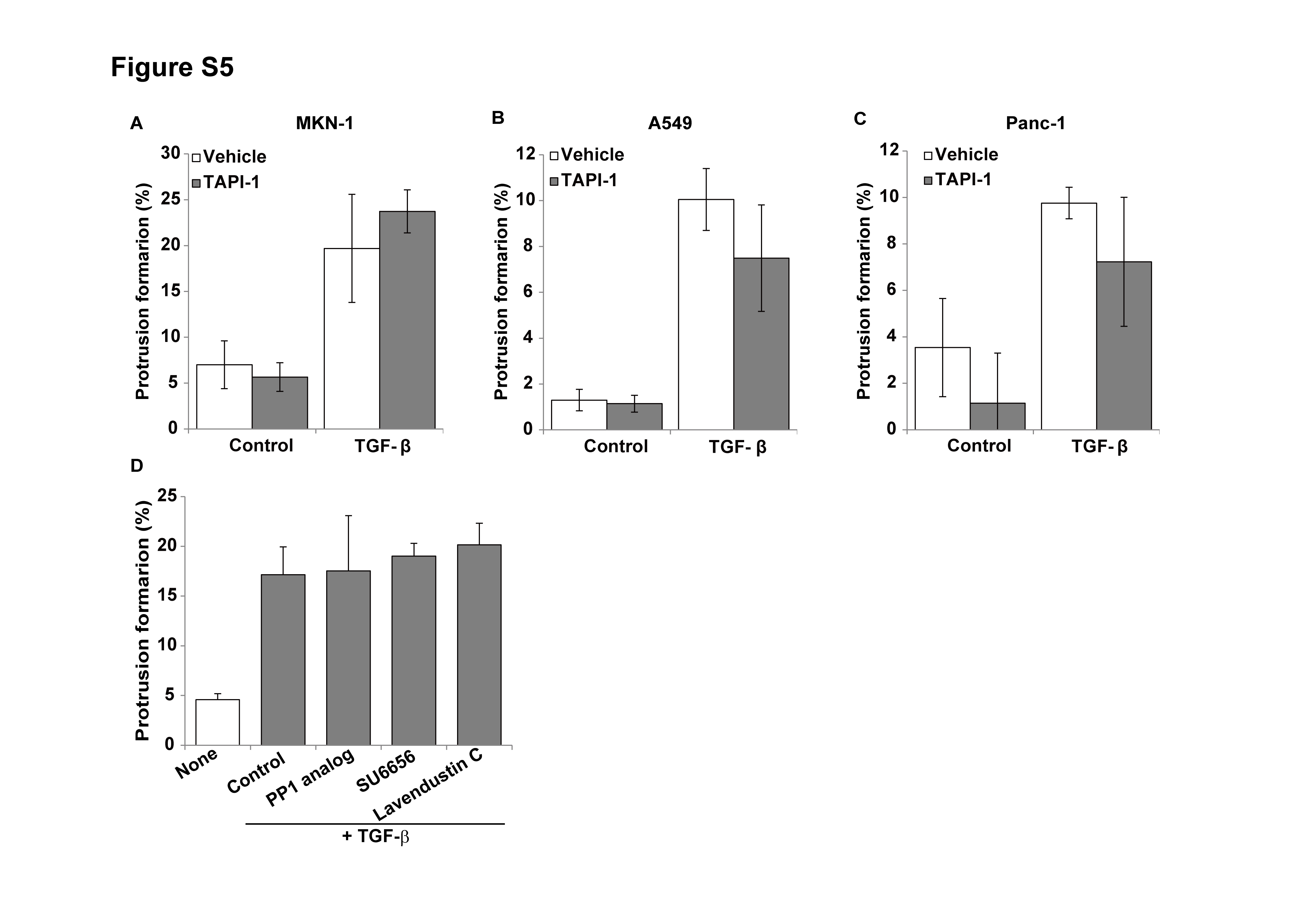

Supplement: Figure S5 — Effects of synthetic MMP inhibitor and signal inhibitors on protrusion formation of MKN-1 cells in 3D collagen gel. MKN-1(A), A549 (B), and Panc-1 (C) cells were incubated without (open columns) or with 10 µM TAPI-1 in the absence (Control) or presence (TGF-ß) of TGF-ß for 5 days, and protrusion formation was quantified. (D) MKN-1 cells were incubated without (None) or with the following inhibitors in the absence (open column) or presence (closed columns) of TGF-ß for 24 h: PP1-analog (1 µM), SU6656 (1 µM), or lavendustin C (1 µM). Other experimental conditions are described in Figures 5 and 7. (TIF) [file pone.0053209.s005.tif]
